# Childhood hearing impairment and fertility in Norway

**DOI:** 10.1038/s41598-021-04195-7

**Published:** 2022-01-10

**Authors:** Vegard Skirbekk, Éric Bonsang, Bo Engdahl

**Affiliations:** 1 Centre for Fertility and Health, Norwegian Institute of Public Health, Postboks 4404 Nydalen, 0403 Oslo, Norway; 2 Columbia University, New York, USA; 3 Université Paris Dauphine, Paris, France; 4 Norwegian Institute of Public Health, Oslo, Norway

**Keywords:** Social evolution, Risk factors, Disability

## Abstract

There is a lack of studies assessing how hearing impairment relates to reproductive outcomes. We examined whether childhood hearing impairment (HI) affects reproductive patterns based on longitudinal Norwegian population level data for birth cohorts 1940–1980. We used Poisson regression to estimate the association between the number of children ever born and HI. The association with childlessness is estimated by a logit model. As a robustness check, we also estimated family fixed effects Poisson and logit models. Hearing was assessed at ages 7, 10 and 13, and reproduction was observed at adult ages until 2014. Air conduction hearing threshold levels were obtained by pure-tone audiometry at eight frequencies from 0.25 to 8 kHz. Fertility data were collected from Norwegian administrative registers. The combined dataset size was N = 50,022. Our analyses reveal that HI in childhood is associated with lower fertility in adulthood, especially for men. The proportion of childless individuals among those with childhood HI was almost twice as large as that of individuals with normal childhood hearing (20.8% vs. 10.7%). The negative association is robust to the inclusion of family fixed effects in the model that allow to control for the unobserved heterogeneity that are shared between siblings, including factors related to the upbringing and parent characteristics. Less family support in later life could add to the health challenges faced by those with HI. More attention should be given to how fertility relates to HI.

## Introduction

There is a scarcity of studies assessing how hearing impairment relates to reproductive outcomes. The effects of early life hearing impairment (HI) on fertility patterns has to our knowledge, never been analyzed using longitudinal population level data with objective measures of hearing. Longitudinal data with hearing observed early in life are necessary to study whether HI influences fertility in adult life. We use unique new data from the School Hearing Investigation in Nord-Trøndelag (SHINT), an audiometric screening of all schoolchildren attending regular schools in the County of Nord-Trøndelag from 1954 to 1986. Following the children for 28–60 years allow us to study how childhood HI relates to male and female childbearing patterns.

The relatively few existing studies of hearing and fertility tend to use relatively small samples. E.g., one regional study in Sweden identified that cohort fertility was only 1.3 children among the deaf population compared to 1.6 children per woman for the general population—this was driven by very high levels of childlessness among the deaf women (affecting more than 4 out of 10)^1^. Another US based study (N = 682 deaf adults and 602 hearing siblings) found that although marital rates were similar by hearing status, married deaf individuals had a lower number of children than married normal hearing individuals (2.1 vs. 2.3 children)^2^. The lack of representative population level studies calls for more inclusive assessments.

### Causes of hearing impairment

Global prevalence estimates of disabling hearing loss in children has been estimated to be 1.7% and in high income countries 0.5%^3^. In children, most HI cases are due to congenital and neonatal conditions^4,5^. Hearing impairment and deafness have been found to be linked to specific genotypes^6^. Infections can also cause hearing impairment^7,8^; hearing loss among children is frequently a result of meningitis, measles, mumps or prenatal conditions including maternal rubella^9^. HI can be a side-effect resulting from other causes certain cancers^10,11^; injuries and traumas to the head^8^ as well as noise exposures, both temporary and long-term^12,13^.

### Implications of hearing loss for partnering and childbearing

Hearing impairment has been found to be associated socioeconomic factors, including school attainment and income^14,15^, and HI may through these factors reduce partnering opportunities for those who are deaf or hearing impaired^16^. The hearing impaired have a greater disease burden and higher mortality^17,18^. Education, income, communication skills, and health affects the likelihood of entering a stable partnerships^20,21^. A stable partnership remains one of the strongest predictors of childbearing in advanced societies^19^.

## Methods

This study investigates whether those with a particular hearing loss during childhood (i.e. prior fertility decisions) have fewer children. If so, we will examine if this is mediated by partnership status or education, and if it has changed over time. A major advantage of our study is that we do not rely on self-assessed hearing. Most existing datasets use subjective HI, which is often a difficult measure with low comparability. Subjective measures of hearing could be poorly related to actual hearing, but rather influenced by peer groups, contextual factors, personality, degree of optimism as well as socioeconomic standards^22^.

### Study population

The relationship between fertility and hearing has not yet been studied using population level data incorporating objective hearing assessments. We combine data from the School Hearing Investigation in Nord-Trøndelag (SHINT), data from the Trøndelag Health Study (HUNT), and population registry data (N = 50,022) which allow these relations to be studied.

Our data comes from the HUNT survey (https://www.ntnu.edu/hunt). All adult participants included in the survey have been informed and given written consent to use data from the child, main and follow-up studies as well as to link data to other registries including patient registries for research purposes. The Norwegian Data Protection Authority has licensed HUNT Research Centre to store and link data collected in all HUNT surveys. All HUNT surveys, and the present nonparticipant study that includes a linkage to patient and population registry data, are approved by The Norwegian Regional Committee for Medical and Health Research Ethics.

The base for our study is a population cohort with participation in two temporally different health examinations. We included participants from SHINT (Aarhus et al. 2015). In short, nearly all 7-, 10- and 13-years old school children in the entire Nord-Trøndelag County underwent screening with pure-tone audiometry from 1954 to 1986. Children with hearing loss at the screening (thresholds ≥ 20 decibel hearing level (dB HL) at three or more frequencies, or ≥ 30 dB HL at one or more frequencies) were invited to an otorhinolaryngologist (ORL) examination and most attended. For instance, from 1954 to 1962, average attendance at the ORL examinations for children with positive screening was 97%^23^. Altogether, 10,269 children took part in the ORL examination, in which 5547 also took part as adults in the later HUNT wave 1, 2 or 3 (1984, 1995 or 2006, respectively)^24^ which was a necessary criterion to link the SHINT data with the National Population Registry.

An extensive effort was made with the aim to include all children born 1940–1980 residing in Nord-Trøndelag in the SHINT survey, though only children who had hearing problems had records included in the dataset^23^. Hence, our analyses are based on the approximation that children with no notification of hearing impairment in the SHINT survey were recorded as normal hearing. We include all HUNT participants who were born in the same period as the SHINT participants as our study group and observe these at HUNT 1, 2 or 3 (1984, 1995 and 2005) (n = 60,261). For subjects born 1954 and later we have information of residence during primary school age, and for subjects born before 1954 we have information of residence at birth. We excluded those born 1954 and later with confirmed residence outside Nord-Trøndelag during primary school age (n = 5671) and those born before 1953 with confirmed residence outside Nord-Trøndelag at birth (n = 4568). This provides us with a dataset of n = 50,022 participants. A limitation with this procedure is that SHINT participants who died, emigrated or refused to participate in HUNT will be excluded and this which may potentially cause selection bias. However, this problem is reduced by participation rates in HUNT (of all inhabitants in Nord-Trøndelag, 89.4% participated in HUNT 1, 69.5% in HUNT 2 and 54.1% in HUNT 3).

Data from SHINT and HUNT were linked with individual level data from the National Population Registry (compiled by Statistics Norway using a unique 11-digit personal identification code assigned to all Norwegian residents). The Regional Committees for Medical and Health Research Ethics approved the study (REK 23178 “HUNT hørsel”). The study met all requirements in accordance with the General Data Protection Regulation (GDPR) and a Data Protection Impact Assessment (DPIA) was conducted.

### Hearing loss measures

Air conduction hearing threshold levels were obtained by pure-tone audiometry at eight frequencies from 0.25 to 8 kHz according to the Norwegian standards at the time^25^. Hearing thresholds were defined as binaural pure-tone average (PTA) of four frequencies (0.5, 1, 2, and 4 kHz) measured in dB HL. Hearing loss was defined as PTA greater than 25 dB HL. SHINT included several follow-ups, and data from the last audiometric test was used including only permanent hearing losses diagnosed with either sensorineural hearing loss, otosclerosis, chronic suppurative otitis media, or permanent hearing loss after recurrent acute otitis media. Subjects with diagnosis yielding temporary hearing loss such as secretory- and acute otitis media was regarded as normal hearing in childhood with hearing threshold equal to zero. We further considered all subjects born between 1940 and 1980 (being in primary school age during SHINT) and not registered with a hearing loss in SHINT as normal hearing in childhood.

### Children ever born

Data on number of children ever born was obtained for the year 2014 from the National Population Registry.

### Partnership status

As mentioned earlier, we investigate whether partnership status is a mediator in the relationship between childhood HI and fertility. First by using a dummy variable that is equal to one if the individual has ever been married and second by using a dummy variable for if the individual has ever been cohabiting. This information is obtained based on the records from the National Population Registry between 1975 and 2014. Cohabiting status was supplied with self-reported data on cohabiting status obtained from HUNT2 (1996–1998).

### Birth cohort

We considered birth cohort (year of birth) as possible factors modifying the association between adult hearing loss and fertility.

### Education

From national education registers, we included information on level of highest education (primary, secondary or tertiary school attainment).

### Confounder adjustments

For adjusted models we selected covariates that might confound the effect of hearing loss on fertility. We collected information on covariates from national registers and from the HUNT 1, 2 and 3 questionnaires.

The adjusted models first include as additional covariates the educational level of the parents. We then include several health-related variables that may be correlated to both fertility outcomes and HI. Missing data of the parent’s education amounted to 9.6% for the mother and 13.3% for the father. This probably because these parents are deceased and thus had no records. These missing values were imputed using median values according to birth year and sex. Missing data on any of the other covariates (9%) were handled by listwise deletion.

#### Analytical approach

Poisson regression models estimate the association between the number of children ever born and HI, expressed as incidence rate ratio (IRR). We use Poisson rather than negative binomial models because we did not find evidence of overdispersion (i.e., the variance of the outcome is not greater than the mean of the outcome). We have also estimated the models by Ordinary Least Squares as a sensitivity analysis estimated with heteroskedasticity-robust error variance and we obtained similar results. The association with childlessness is estimated by a logit model and expressed as odds ratio (OR). The latter was estimated with robust error variance.

As a robustness check, we also estimated family fixed effect (FFE) Poisson and logit models. FFE models analyze fertility as a function of features that vary between siblings. The models control for all observed and non-observed confounders that are shared between siblings, including factors related to upbringing and parent characteristics. The drawback is that this analysis is restricted to individuals with at least one sibling (of any sex). The family fixed effects were compared with estimates from ordinary Poisson and logit models in the same restricted sample of siblings. We further tested if the effect differed according to severity of hearing loss by dividing childhood HI into three categories: moderate-severe (> 40 dB HL) (n = 161), mild (26–40 dB HL) (n = 410) and slight hearing loss (16–25 dB HL) (n = 489).

All analyzes were done separately for men and women adjusting for birth year and subsequently also accounting for other covariates.

In order to study if the associations were mediated by cohabiting status or education, we applied a counterfactual approach allowing us to estimate mediation effects in a non-linear model when exposure-mediator interaction is present^26^. The proportion mediated was calculated as OR_NDE_(OR_NIE_ − 1)/(OR_NDE_ × OR_NIE_ − 1) in which OR_NDE_ is the odds ratio for the natural direct effect and OR_NIE_ is the risk ratio for the natural indirect effect.

Potential birth cohort trends were estimated by stratifying on birth cohorts.

All methods were performed in accordance with relevant regulations from the data owners.

## Results

### Descriptive statistics

Table 1 presents the descriptive statistics of the analytical sample. Among the 50,022 subjects in the childhood hearing loss cohort, 571 subjects had a childhood hearing loss. As expected, individuals with childhood HI have fewer children than those with childhood normal hearing. The difference is statistically significant at the 1 percent-level and is more salient among men than women and is even more striking when we look at the percentage of individuals being childless. The proportion of childless individuals among those with childhood HI is almost twice as large as that of individuals with normal childhood hearing (20.8% vs. 10.7%). We also note that individuals with childhood HI are less likely to have ever been married.

**Table 1:** Descriptive statistics.

Variable		All subjects	Childhood hearing loss		p-value^d^
			No	Yes	
		n = 50,022	n = 49,451	n = 571	
All	Age, mean (SD)	56.3 (10.6)	56.3 (10.6)	55.2 (9.2)	0.0146
	Number of children^a^, mean (SD)	2.2 (1.2)	2.2 (1.2)	1.9 (1.3)	< 0.0001
	Childless^a^, N (%)	5406 (10.8)	5287 (10.7)	119 (20.8)	< 0.0001
	Ever married^b^, N (%)	38,665 (77.3)	38,275 (77.4)	390 (68.3)	< 0.0001
	Ever cohabit^c^, N (%)	46,552 (93.1)	46,071 (93.2)	481 (84.2)	< 0.0001
	**Education, N (%)**				
	Primary	9748 (19.5)	9609 (19.4)	139 (24.3)	
	Secondary	26,622 (53.2)	26,303 (53.2)	319 (55.9)	
	Tertiary	13,652 (27.3)	13,539 (27.4)	113 (19.8)	< 0.0001
Female	Age, mean (SD)	55.8 (10.7)	55.8 (10.7)	54.9 (9.7)	0.2075
	Number of children^a^, mean (SD)	2.3 (1.1)	2.3 (1.1)	2.2 (1.2)	0.0394
	Childless^a^, N (%)	1720 (7.0)	1697 (6.9)	23 (11.2)	0.017
	Ever married^b^, N (%)	19,802 (80.1)	19,650 (80.2)	152 (73.8)	0.022
	Ever cohabit^c^, N (%)	23,493 (95.2)	23,308 (95.1)	185 (89.8)	< 0.0001
	Education, N (%)				
	Primary	4944 (20.0)	4891 (20.0)	53 (25.7)	
	Secondary	11,803 (47.8)	11,696 (47.7)	107 (51.9)	
	Tertiary	7968 (32.2)	7922 (32.3)	46 (22.3)	0.005
Male	Age, mean (SD)	56.7 (10.4)	56.8 (10.4)	55.4 (8.8)	0.0125
	Number of children^a^, mean (SD)	2.1 (1.3)	2.1 (1.3)	1.8 (1.3)	< 0.0001
	Childless^a^, N (%)	3686 (14.6)	3590 (14.4)	96 (26.3)	< 0.0001
	Ever married^b^, N (%)	18,863 (74.5)	18,625 (74.7)	238 (65.2)	< 0.0001
	Ever cohabit^c^, N (%)	22,998 (91.4)	22,704 (91.0)	294 (80.5)	< 0.0001
	**Education, N (%)**				
	Primary	4804 (19.0)	4718 (18.9)	86 (23.6)	
	Secondary	14,819 (58.6)	14,607 (58.6)	212 (58.1)	
	Tertiary	5684 (22.5)	5617 (22.5)	67 (18.4)	0.032

Hearing thresholds were defined as binaural pure-tone average (PTA) of four frequencies (0.5, 1, 2, and 4 kHz) measured in dB HL. Childhood Hearing loss was defined as PTA > 25 dB HL, moderate-severe profound hearing loss as PTA of > 40 dB HL, mild hearing loss as PTA 26–40 dB HL and slight hearing loss as PTA 16–25 dB HL. Only children diagnosed with permanent sensorineural hearing loss were included. Altogether 571 persons were classified with childhood hearing loss, 161 with moderate-severe profound sensorineural hearing loss, 410 with mild sensorineural hearing loss, and 489 with slight sensorineural hearing loss (see further details under “measures”).

^a^Fertility observed in 2014.

^b^Marital status = 'Ever married' at least once in the period from 1975 to 2014.

^c^Ever cohabit = 'Ever cohabit' at least once in the period from 1975 to 2014.

^d^T-test for means and Chi-Square test for frequencies.

### Main results

Table 2 shows the association between childhood HI and fertility estimated by a Poisson regression model. It suggests that childhood hearing loss is associated with a lower number of children, but the association is significantly different from zero at the 5 percent-level only for men but not for women. The inclusion of the different control variables in the model does not significantly affect the estimate of the parameter of interest: the number of children of men with childhood HI is 18% lower (95% CI − 10 to − 26%) than for men with normal hearing, while it is 7% lower for women (95% CI 3 to − 17%). The predicted number of children ever born is 1.79 (95% CI 1.64 to 1.93) for childhood HI vs 2.12 (95% CI 2.11 to 2.15) for normal hearing in men and 2.19 (95% CI 1.98 to 2.40) children for HI vs 2.35 (95% CI 2.33 to 2.37) children for normal hearing in women.

**Table 2:** The relation of childhood hearing loss to the number of children ever born.

		IRR	[95% CI]	AME	[95% CI]	SE	[95% CI]	p-value
Model 1	All^a^	0.88	0.83, 0.93	− 0.29	− 0.42, − 0.16	− 0.13	− 0.19, − 0.07	0.000
	Female	0.94	0.85, 1.03	− 0.16	− 0.37, 0.06	− 0.07	− 0.16, 0.03	0.158
	Male	0.84	0.78, 0.91	− 0.36	− 0.52, − 0.20	− 0.17	− 0.25, − 0.09	0.000
Model 2	All^a^	0.88	0.83, 0.93	− 0.32	− 0.45, − 0.19	− 0.14	− 0.20, − 0.08	0.000
	Female	0.93	0.85, 1.03	− 0.16	− 0.38, 0.06	− 0.07	− 0.16, 0.02	0.152
	Male	0.84	0.78, 0.91	− 0.36	− 0.52, − 0.19	− 0.17	− 0.25, − 0.09	0.000
Model 3	All^a^	0.87	0.82, 0.93	− 0.30	− 0.44, − 0.16	− 0.14	− 0.20, − 0.07	0.000
	Female	0.93	0.85, 1.03	− 0.16	− 0.37, 0.05	− 0.07	− 0.17, 0.03	0.154
	Male	0.84	0.77, 0.91	− 0.38	− 0.55, − 0.21	− 0.18	− 0.26, − 0.10	0.000

*IRR* incidence rate ratio, *AME* average marginal effect (change in number of children), *SE* Semi-elasticity (proportional change in number of children).

Model 1—adjusted for age.

Model 2—adjusted for age, mother and father's education.

Model 3—adjusted for age, mother and father's education and self-reported health, systolic and diastolic blood pressure, motor, visual, somatic and mental impairment, and smoking.

^a^Also adjusted for sex.

Table 3 presents the association between childhood HI and childlessness estimated by a logit model. As suggested by Table 1, it shows that childhood HI is positively and significantly associated with a higher probability to be childless, especially among men. For them, the probability to be childless increases by 9 percentage points (95% CI 6 to 12%) when they suffered from childhood HI. Compared to a sample mean of 14.6% (see Table 1), this difference is large in magnitude. For women, the association is also positive and significant at the 5% level but it becomes only non-significant at the 10% level in the third model only, suggesting the evidence for the presence of an effect of childhood HI on the probability to be childless for women is driven by confounding factors.

**Table 3:** The relationship of childhood hearing loss to childlessness.

		OR	[95% CI]	AME	[95% CI]	SE	[95% CI]	p-value
Model 1	All^a^	1.98	1.61–2.43	0.064	0.045–0.084	0.61	0.42–0.79	0.000
	Female	1.67	1.08–2.59	0.033	0.005–0.061	0.48	0.07–0.88	0.021
	Male	2.08	1.65–2.64	0.091	0.062–0.120	0.63	0.43–0.83	0.000
Model 2	All^a^	1.98	1.61–2.43	0.064	0.045–0.084	0.61	0.42–0.79	0.000
	Female	1.71	1.10–2.65	0.035	0.006–0.063	0.50	0.09–0.91	0.016
	Male	2.08	1.64–2.63	0.090	0.061–0.120	0.62	0.42–0.83	0.000
Model 3	All^a^	1.99	1.60–2.47	0.061	0.042–0.081	0.62	0.42–0.81	0.000
	Female	1.49	0.92–2.39	0.024	− 0.005–0.052	0.37	− 0.08–0.82	0.104
	Male	2.17	1.70–2.78	0.092	0.063–0.121	0.67	0.45–0.88	0.000

*OR* Odds ratio, *AME* average marginal effect (average change in probability of being childless), *SE* Semi-elasticity (proportional change in probability of being childless).

Model 1—adjusted for age.

Model 2—adjusted for age, mother and father's education.

Model 3—adjusted for age, mother and father's education and self-reported health, systolic and diastolic blood pressure, motor, visual, somatic and mental impairment, and smoking.

^a^Also adjusted for sex.

### Mediation analyses

The results of the mediation analysis (see Table 4) suggest that 47% of the association is mediated by marriage for both men and women, and this result holds also when controlling for confounders. Marriage and cohabiting account for 51% of the association for women and 65 for men in model 3. Finally, Table 4 shows that the role of education in explaining the difference in the number of children between the normal hearing childhood and the childhood HI individuals is rather small: the results using the model 3 suggest that it only explains 9% of the difference for women and 6% for men when compared to model 1. The results of mediation analysis (see Supplementary Table 1) regarding the probability to be childless show a similar pattern but there are some differences. First, the fact of cohabiting or being married seems to play a large role in the association between the probability to be childless and childhood HI. It accounts for 64% of the difference for women and 85% for men in the model 3.

**Table 4:** Childhood hearing loss and number of children ever born. Proportion mediated by partnership status, education and health.

		Married	Married or cohabit	Education
Model 1	All^a^	0.47	0.62	0.13
	Female	0.47	0.59	0.25
	Male	0.47	0.64	0.09
Model 2	All^a^	0.47	0.62	0.11
	Female	0.44	0.60	0.20
	Male	0.47	0.65	0.08
Model 3	All^a^	0.44	0.60	0.07
	Female	0.41	0.51	0.09
	Male	0.44	0.65	0.06

Model 1—adjusted for age.

Model 2—adjusted for age, mother’s and father's education.

Model 3—adjusted for age, mother’s and father's education and self-reported health, systolic and diastolic blood pressure, motor, visual, somatic and mental impairment, and smoking.

^a^Also adjusted for sex.

### Cohort effects

Finally, to explore if the associations differ across cohorts, we estimated the models for two cohorts: those born between 1940 and 1959 and those born between 1960 and 1980. The results presented in Supplementary Table 2 (for the number of children) and Supplementary Table 3 (for childlessness) suggest that the association between fertility and childhood HI is higher among later born male cohorts. For women, the association between childhood HI and the number of children is not significantly different from zero. Regarding childlessness (Supplementary Table 3), the association is significantly different from zero at the 5%-level for women born between 1960 and 1980 but not for the 1940–1959 cohort.

### Fertility and the severity of childhood HI

We complement our main results by investigating whether the severity of childhood HI also play a role in fertility outcomes by dividing the sample into four categories as described above: Individuals with moderate to severe childhood HI, those with mild childhood HI, those with slight childhood HI, and those with no childhood HI. Supplementary Table 4 shows that fertility is not related to childhood HI for women whatever the level of severity of the HI. Among men, we clearly observe a dose–response relationship: the more severe the childhood HI, the lower the number of children. Regarding the results for childlessness (see Supplementary Table 5), a similar pattern is observed for men (the more severe the HI, the higher the likelihood to be childless). For women, only women suffering from mild-to-severe HI are significantly more likely to be childless.

### Robustness checks

In this section, we report the results of our robustness checks by estimating family fixed effect (FFE) Poisson and logit models that allow us to further control for family-invariant unobserved heterogeneity for individuals with at least one sibling. The results regarding the number of children are displayed in Supplementary Table 6 while Supplementary Table 7 shows the results regarding the probability of childlessness. These tables also show the results of the models that do not include FFE based on the same sample. It shows that our results are robust to this new model exploiting variation in childhood HI across siblings. It confirms that the relationship among women is not significantly different from zero at any conventional level while it is significant at the 5% level for men, both regarding the number of children and the probability to be childless. Note nevertheless that the relationship between childhood HI and the probability to be childless (also regarding the number of children) becomes smaller in magnitude once we control for FFE for women. It suggests that unobserved family heterogeneity is positively correlated with the probability to have HI during childhood and the probability to be childless for women, but not for men. Nevertheless, the difference in the estimates across models are modest in magnitude and the confidence intervals considerably overlap.

## Discussion

The current study discussed how fertility relates to HI using population level longitudinal data with objective hearing assessments. Our results suggest that (i) individuals with HI have fewer children than their normal hearing peers, and that this relationship is only observed for men, and (ii) this is to a large extent explained by a lower likelihood of having ever been married or cohabited.

Our thresholds analysis for childhood HI shows that it does not only affect individuals with severe childhood HI but also those with mild childhood HI and is therefore relevant for a broader section of the population rather than only those with severe HI or deafness, although the effects are stronger among those with more severe HI.

This study is conducted using larger longitudinal data than what has previously been available. Our findings remain significant also when controlling for family fixed effects. Moreover, many earlier studies have been based on self-reported hearing, which is a measure that can be difficult to interpret (e.g., individuals may compare themselves to their peer groups rather than to fixed standards). We use audiometrically based measures of hearing function, which gives objective assessments.

While this study provides strong evidence suggesting that HI affects fertility, we cannot rule out the possibility that our results are biased due to sample selection or unobserved confounders. Sample selection bias might be due to selected mortality or selected emigration. Assuming that mortality is positively correlated with childhood HI and negatively correlated with fertility, bias due to selected mortality would imply that our estimated association is underestimating the negative effect of childhood HI on fertility. The bias due to selected emigration is expected to overestimate the negative effect of childhood HI on fertility if emigration is negatively correlated with childhood HI and positively correlated with fertility. Nevertheless, these biases are likely to be very small given relatively few deaths or emigration occur before HUNT 1 (1986) in the relevant age groups in Norway^27^. Further, we lack information on the cognitive ability of the participants in our study—and as a substantial share of Norwegian children with hearing loss may have cognitive challenges^28^, which could be a source of bias. Nevertheless, the model using family fixed effects allows to control for a large share of the unobserved heterogeneity. Moreover, thanks to the longitudinal nature of the data, reverse causality cannot explain the observed association.

Other health characteristics may potentially help explain why the individuals with HI have lower fertility, such as possible social, psychological or cognitive characteristics that we do not observe in our data and therefore are unable to control for. Nevertheless, HUNT includes a very broad array of health, economic and sociodemographic information and linkage to national registers. Moreover, HUNT is one of the largest health surveys in the world—and has a relatively high response rate (ranging between 54 and 89%) compared to most of health survey of similar size (E.g., the response rate is at 5.5% for UK-biobank). As we also have national register data, hence we are able to assess the representatively of the HUNT sample compared to the non-respondents.

The stronger negative effect of HI on male as opposed to female fertility could be linked to sex differences in partnering selection. The fact that male income remains a stronger determinant of partnership patterns while female income has little effect^21,29^ may affect childbearing outcomes between men and women with HI.

Societal and technological developments have changed the life of many hearing impaired in recent decades, where several improvements in hearing aids or change in communication form (online text-based communications) benefit individuals with HI. In spite of this, our study shows that HI is strongly negative related to fertility outcomes.

This study has relevance to understanding health outcomes among those with hearing impairment. As parenthood, marital status and family support is a strong determinant of health^30–32^, the high prevalence of low fertility and childlessness among the hearing impaired could adverse relate to health risk factors associated with greater risk of lifestyle related illnesses and shorter life expectancy. One may consider providing more emphasis to adverse lifestyles associated with a lack of family as a health challenge among the hearing impaired.

## Supplementary Information

Supplementary Tables.
