# Supplementary material for: Childhood hearing impairment and fertility in Norway
Source: Sci Rep. 2022 Jan 10;12:402. doi: 10.1038/s41598-021-04195-7 (PMC8748703; doi:10.1038/s41598-021-04195-7)
Supplement: Supplementary file 1 — Supplementary Tables. [file 41598_2021_4195_MOESM1_ESM.docx]

| **Supplemental materials**  **Supplemental table 1. Childhood hearing loss and being childless. Proportion mediated by marriage, cohabitation status and education.** | | | | |
| --- | --- | --- | --- | --- |
|  |  | Married | Married or cohabit | Education |
| Model 1 | All^1^ | 0.38 | 0.72 | 0.18 |
|  | Female | 0.42 | 0.69 | 0.44 |
|  | Male | 0.37 | 0.79 | 0.11 |
| Model 2 | All^1^ | 0.38 | 0.73 | 0.16 |
|  | Female | 0.39 | 0.70 | 0.37 |
|  | Male | 0.37 | 0.80 | 0.10 |
| Model 3 | All^1^ | 0.38 | 0.73 | 0.14 |
|  | Female | 0.51 | 0.64 | 0.41 |
|  | Male | 0.35 | 0.85 | 0.09 |
| Model 1 - adjusted for age | | |  |  |
| Model 2 - adjusted for age, mother and father's education | | | | |
| Model 3 - adjusted for age, mother and father's education and self-reported health, systolic & diastolic blood pressure, motor, visual, somatic & mental impairment, and smoking  ^1^ Also adjusted for sex | | | | |

| **Supplemental table 2. The relationship between childhood hearing loss and the number of children by birth cohort.** | | | | | | | | | | | | | | | |
| --- | --- | --- | --- | --- | --- | --- | --- | --- | --- | --- | --- | --- | --- | --- | --- |
|  | Birth cohort | IRR | | [95% CI] | | AME | | [95% CI] | | SE | | [95% CI] | | p-value | |
| All | 1940-1959 | 0.90 | | 0.83,0.98 | | -0.22 | | -0.40,-0.03 | | -0.10 | | -0.18,-0.01 | | 0.013 | |
|  | 1960-1980 | 0.85 | | 0.77,0.93 | | -0.36 | | -0.55,-0.17 | | -0.17 | | -0.26,-0.08 | | 0.000 | |
| Female | 1940-1959 | 0.95 | | 0.84,1.09 | | -0.09 | | -0.39,0.22 | | -0.04 | | -0.16,0.09 | | 0.481 | |
|  | 1960-1980 | 0.93 | | 0.80,1.07 | | -0.21 | | -0.52,0.10 | | -0.09 | | -0.23,0.04 | | 0.297 | |
| Male | 1940-1959 | 0.87 | | 0.78,0.97 | | -0.30 | | -0.53,-0.07 | | -0.14 | | -0.24,-0.03 | | 0.010 | |
|  | 1960-1980 | 0.79 | | 0.70,0.89 | | -0.45 | | -0.68,-0.21 | | -0.22 | | -0.34,-0.11 | | 0.000 | |
| IRR-incidence rate ratio | | |  | |  | |  | |  | |  | |  | |  |
| AME - average marginal effect (change in number of children) | | | | | | | | |  | |  | |  | |  |
| SE - Semi-elasticity (proportional change in number of children) | | | | | | | | |  | |  | |  | |  |
| Adjusted for age, mother and father's education and self-reported health, systolic & diastolic blood pressure, motor, visual, somatic & mental impairment, and smoking | | | | | | | | | | | | | | | |
| ^1^ Also adjusted for sex | | |  | |  | |  | |  | |  | |  | |  |

| **Supplemental table 3. The relationship of childhood hearing loss to childlessness by birth cohort.** | | | | | | | | | | | | | |
| --- | --- | --- | --- | --- | --- | --- | --- | --- | --- | --- | --- | --- | --- |
|  | Birth cohort | OR | | [95% CI] | | AME | | [95% CI] | | | SE | [95% CI] | p-value |
| All^1^ | 1940-1959 | 1.87 | | 1.36,2.56 | | 0.053 | | 0.026,0.079 | | | 0.55 | 0.27,0.82 | 0.000 |
|  | 1960-1980 | 2.13 | | 1.57,2.87 | | 0.080 | | 0.051,0.110 | | | 0.68 | 0.43,0.92 | 0.000 |
| Female | 1940-1959 | 1.50 | | 0.73,3.06 | | 0.025 | | -0.014,0.064 | | | 0.40 | -0.21,1.01 | 0.270 |
|  | 1960-1980 | 1.43 | | 0.75,2.72 | | 0.042 | | 0.001,0.084 | | | 0.55 | 0.01,1.09 | 0.274 |
| Male | 1940-1959 | 1.96 | | 1.37,2.79 | | 0.074 | | 0.035,0.113 | | | 0.58 | 0.27,0.88 | 0.000 |
|  | 1960-1980 | 2.44 | | 1.73,3.45 | | 0.116 | | 0.071,0.162 | | | 0.69 | 0.42,0.96 | 0.000 |
| OR-Odds ratio | | |  | |  | |  | |  |  | |  |  |
| AME - average marginal effect (average change in probability of being childless) | | | | | | | | | | | | | |
| SE - Semi-elasticity (proportional change in probability of being childless) | | | | | | | | | |  | |  |  |
| Adjusted for age, mother and father's education and self-reported health, systolic & diastolic blood pressure, motor, visual, somatic & mental impairment, and smoking | | | | | | | | | | | | | |
| ^1^ Also adjusted for sex | | |  | |  | |  | |  |  | |  |  |

| **Supplemental table 4. The relationship of level of childhood hearing loss to children ever born** | | | | | | | | | | | | | | | |  |
| --- | --- | --- | --- | --- | --- | --- | --- | --- | --- | --- | --- | --- | --- | --- | --- | --- |
|  | Hearing loss | | IRR | [95% CI] | | AME | | [95% CI] | | SE | | [95% CI] | | p-value | |  |
| All^1^ | Slight | | 0.96 | 0.91,1.03 | | -0.08 | | -0.22,0.06 | | -0.04 | | -0.10,0.03 | | 0.265 | |  |
|  | Mild | | 0.88 | 0.82,0.95 | | -0.26 | | -0.40,-0.12 | | -0.12 | | -0.20,-0.05 | | 0.001 | |  |
|  | Moderate-severe | | 0.84 | 0.75,0.95 | | -0.35 | | -0.58,-0.13 | | -0.17 | | -0.29,-0.05 | | 0.005 | |  |
| Female | Slight | | 0.99 | 0.90,1.09 | | -0.02 | | -0.24,0.20 | | -0.01 | | -0.10,0.09 | | 0.863 | |  |
|  | Mild | | 0.93 | 0.83,1.04 | | -0.17 | | -0.42,0.07 | | -0.08 | | -0.19,0.04 | | 0.186 | |  |
| Male | Moderate-severe | | 0.95 | 0.78,1.14 | | -0.13 | | -0.54,0.29 | | -0.06 | | -0.24,0.13 | | 0.564 | |  |
|  | Slight | | 0.94 | 0.86,1.03 | | -0.12 | | -0.30,0.05 | | -0.06 | | -0.15,0.03 | | 0.184 | |  |
|  | Mild | | 0.86 | 0.78,0.94 | | -0.31 | | -0.48,-0.14 | | -0.16 | | -0.25,-0.06 | | 0.001 | |  |
|  | Moderate-severe | | 0.79 | 0.67,0.92 | | -0.46 | | -0.72,-0.19 | | -0.24 | | -0.40,-0.08 | | 0.003 | |  |
| IRR-incidence rate ratio | | |  |  | |  | |  | |  | |  | |  | |  |
| AME - average marginal effect (change in number of children) | | | | | | | | | | | | | | | |  |
| SE - Semi-elasticity (proportional change in number of children) | | | | | | | | | |  | |  | |  | |  |
| Adjusted for age, mother and father's education and self-reported health, systolic & diastolic blood pressure, motor, visual, somatic & mental impairment, and smoking | | | | | | | | | | | | | | | | |
| ^1^ Also adjusted for sex | |  | | |  | |  | |  | |  | |  | |  | |

| **Supplemental table 5. The relationship between level of childhood hearing loss to childlessness** | | | | | | | | |
| --- | --- | --- | --- | --- | --- | --- | --- | --- |
|  | Hearing loss | OR | [95% CI] | AME | [95% CI] | SE | [95% CI] | p-value |
| All^1^ | Slight | 1.10 | 0.82,1.48 | 0.01 | -0.02,0.04 | 0.09 | -0.18,0.35 | 0.522 |
|  | Mild | 1.72 | 1.32,2.25 | 0.06 | 0.02,0.09 | 0.47 | 0.25,0.70 | 0.000 |
|  | Moderate-severe | 2.75 | 1.89,4.00 | 0.13 | 0.07,0.19 | 0.85 | 0.56,1.14 | 0.000 |
| Female | Slight | 0.98 | 0.54,1.78 | 0.00 | -0.04,0.03 | -0.02 | -0.57,0.54 | 0.955 |
|  | Mild | 1.10 | 0.59,2.08 | 0.01 | -0.03,0.05 | 0.09 | -0.50,0.68 | 0.762 |
|  | Moderate-severe | 2.59 | 1.22,5.51 | 0.08 | -0.01,0.18 | 0.85 | 0.21,1.50 | 0.014 |
| Male | Slight | 1.14 | 0.81,1.60 | 0.02 | -0.03,0.06 | 0.11 | -0.17,0.40 | 0.445 |
|  | Mild | 1.96 | 1.45,2.63 | 0.10 | 0.05,0.15 | 0.55 | 0.32,0.77 | 0.000 |
|  | Moderate-severe | 2.78 | 1.80,4.31 | 0.17 | 0.08,0.26 | 0.80 | 0.50,1.11 | 0.000 |
| OR-Odds ratio | |  |  |  |  |  |  |  |
| AME - average marginal effect (change in proportionality of being childless) | | | | | | | | |
| SE - Semi-elasticity (proportional change in proportionality of being childless) | | | | | | | | |
| Adjusted for age, mother and father's education and self-reported health, systolic & diastolic blood pressure, motor, visual, somatic & mental impairment, and smoking | | | | | | | | |
| ^1^ Also adjusted for sex | |  |  |  |  |  |  |  |

| **Supplemental table 6. The relationship between childhood hearing loss and number of children ever born. Estimates for sibling data. Family fixed effect poisson model and ordinary poisson model.** | | | | | | | | | |
| --- | --- | --- | --- | --- | --- | --- | --- | --- | --- |
|  |  | IRR | | [95% CI] | | SE | [95% CI] | | p-value |
| All^1^ | Family fixed | 0.89 | | 0.81, 0.98 | | -0.12 | -0.21,-0.02 | | 0.013 |
|  | Ordinary | 0.87 | | 0.81, 0.94 | | -0.14 | -0.21,-0.07 | | 0.000 |
| Female | Family fixed | 0.97 | | 0.80, 1.18 | | -0.03 | -0.23,0.16 | | 0.745 |
|  | Ordinary | 0.92 | | 0.82, 1.03 | | -0.09 | -0.20,0.03 | | 0.133 |
| Male | Family fixed | 0.85 | | 0.73, 1.00 | | -0.16 | -0.31,0.00 | | 0.045 |
|  | Ordinary | 0.85 | | 0.77, 0.93 | | -0.17 | -0.26,-0.07 | | 0.000 |
| IRR-incidence rate ratio | | | | | | | | | |
| SE - Semi-elasticity (proportional change in number of children) | | | | | | | |  | |
| Adjusted for age, self-reported health, systolic & diastolic blood pressure, motor, visual, somatic & mental impairment, and smoking | | | | | | | | | |
| ^1^ Also adjusted for sex | | |  |  |  | | |  | |

We estimated family fixed effect (FFE) Poisson and logit models using the *xtpoisson* and *xtlogit* commands in Stata. FFE models analyze fertility as a function of features that vary between siblings. The models control for all observed and non-observed confounders that are shared between siblings, including factors related to upbringing and parent characteristics. The drawback is that they only consider families with at least two children. The family fixed effects were compared with estimates from ordinary poisson and logit models in the same restricted sample of siblings.

Semi-elasticity was estimated directly as the estimated regression coefficient in the FFE-poisson models and in the FFE-logit models by the aextlogit module in Stata ^1,2^.

| **Supplemental table 7. The relationship between childhood hearing loss and childlessness. Estimates for sibling data. Family fixed effect logistic model and ordinary logit model.** | | | | | | | | | | |
| --- | --- | --- | --- | --- | --- | --- | --- | --- | --- | --- |
|  | |  | OR | [95% CI] | | | SE | [95% CI] | | p-value |
| All^1^ | Family fixed | | 1.71 | 1.13-2.57 | | | 0.48 | 0.11-0.85 | | 0.011 |
|  | Ordinary | | 1.93 | 1.50-2.50 | | | 0.59 | 0.36-0.82 | | 0.000 |
| Female | Family fixed | | 1.46 | 0.39-5.48 | | | 0.36 | -0.89-1.60 | | 0.575 |
|  | Ordinary | | 1.69 | 0.95-2.98 | | | 0.49 | -0.04-1.02 | | 0.072 |
| Male | Family fixed | | 2.01 | 1.12-3.58 | | | 0.60 | 0.10-1.10 | | 0.019 |
|  | Ordinary l | | 2.01 | 1.51-2.68 | | | 0.60 | 0.35-0.85 | | 0.000 |
| OR-Odds ratio | | |  | |  |  | | |  | |
| SE - Semi-elasticity (proportional change in probability of being childless) | | | | | | | | | | |
| Adjusted for age, self-reported health, systolic & diastolic blood pressure, motor, visual, somatic & mental impairment, and smoking | | | | | | | | | | |
| ^1^ Also adjusted for sex | | |  | |  |  | | |  | |

References

33 Kitazawa, Y. Hyperbolic transformation and average elasticity in the framework of the fixed effects logit model. (2012).

34 Santos Silva, J. AEXTLOGIT: Stata module to compute average elasticities for fixed effects logit. (2019).

1 Kitazawa, Y. Hyperbolic transformation and average elasticity in the framework of the fixed effects logit model. *Theoretical Economics Letters* **2**, 192-199 (2012).

2 Santos Silva, J. AEXTLOGIT: Stata module to compute average elasticities for fixed effects logit. *Statistical Software Components S458254, Boston College Department of Economics*  (2020).
